# Differential Relationship between Tobacco Control Policies and U.S. Adult Current Smoking by Poverty

**DOI:** 10.3390/ijerph16214130

**Published:** 2019-10-26

**Authors:** Lauren M. Dutra, Matthew C. Farrelly, James Nonnemaker, Brian Bradfield, Jennifer Gaber, Minal Patel, Elizabeth C. Hair

**Affiliations:** 1 Center for Health Policy Science and Tobacco Research, RTI International, 3040 East Cornwallis Road, Durham, NC 27709, USA; mcf@rti.org (M.C.F.); jnonnemaker@rti.org (J.N.); bbradfield@rti.org (B.B.); jgaber@rti.org (J.G.); 2 Schroeder Institute, Truth Initiative, 900 G Street Northwest, Fourth Floor, Washington, DC 20001, USA; mpatel@truthinitiative.org (M.P.); ehair@truthinitiative.org (E.C.H.)

**Keywords:** smoking, cigarettes, poverty, policy, tax, tobacco control funding, clean indoor air laws, smoke-free air laws

## Abstract

The study’s purpose was to identify differences in the relationship between tobacco control policies and smoking by poverty. We matched state smoke-free air law coverage (SFALs), tobacco control funding (TCF), and cigarette taxes with individual current smoking and demographics from supplements to the Current Population Survey (1985–2015). We regressed (logistic) smoking on policy variables, poverty (<138% of poverty line versus ≥138% of poverty line), interactions of policy and poverty, and covariates, presenting beta coefficients instead of odds ratios because it is difficult to interpret interactions using odds ratios (they are ratios of odds ratios). We coded SFALs as (1) proportion of state covered by 100% workplace, restaurant and bar laws (SFAL-All) or (2) proportion of state covered by workplace laws (SFAL-WP) and proportion covered by restaurant or bar laws (SFAL-RB). In the SFAL-All model, SFAL-All (Beta coeff: −0.03, 95% CI: −0.06, −0.002), tax (Coeff: −0.06, 95% CI: −0.07, −0.05), and TCF (Coeff: −0.01, 95% CI: −0.01, −0.001) were associated with less smoking. In this model, the interaction of SFAL-All by poverty was significant (Coeff: 0.08, 95% CI: 0.02, 0.13). In the SFAL-WP/RB model, SFAL-RB (Coeff: −0.05, 95% CI: −0.08, −0.02), tax (Coeff: −0.05, 95% CI: −0.06, −0.04), and TCF (Coeff: −0.01, 95% CI: −0.01, −0.00) were significant. In the same model, SFAL-WP (Coeff: 0.09, 95% CI: 0.03, 0.15), SFAL-RB (Coeff: −0.14, 95% CI: −0.19, −0.09), and TCF (Coeff: 0.01, 95% CI: 0.00, 0.02) interacted with poverty. Tax by poverty was of borderline significance in this model (Coeff = 0.02, 95% CI: −0.00, 0.04, *p* = 0.050). Among adults, SFALs, TCF, and tax were associated with less current smoking, and SFALs and TCF had differential relationships with smoking by poverty.

## 1. Introduction

Each year, cigarette smoking accounts for over 4,800,000 deaths among US adults, and a disproportionate portion of these deaths occurs among members of vulnerable groups, such as low-income individuals [1]. Low-income individuals have a higher prevalence of cigarette smoking than high-income individuals [1,2]. On average, they start smoking earlier and tend to smoke for more years over their lifetimes [3]. They also have more difficulty quitting smoking [1], are less likely to have access to healthcare, and have higher exposure to tobacco advertising [3]. As a result, low-income smokers have high tobacco-related disease and death rates, in part, due to environmental and occupational risk factors for tobacco-related diseases [4], such as lack of coverage by smoke-free air laws (SFALs; also known as clean indoor air laws) [5]. SFALs and other policies, such as taxes and state tobacco control funding (TCF), have proven effective for reducing smoking overall and may narrow income-based smoking disparities [1].

Low-income smokers are more likely to be exposed to SHS due to working in hospitality establishments, such as bars and restaurants, and may be more likely than high-income smokers to benefit from the introduction of strong, effectively enforced SFALs [6,7,8]. Because they may be more price sensitive than high-income smokers [9], higher taxes and minimum prices for tobacco products may be more effective at reducing cigarette smoking [10,11] among low-income youth and adults [12,13,14,15,16,17,18,19]. Per capita tobacco control spending, which supports quitlines, media campaigns, community mobilization for tobacco control policies, and tobacco use surveillance and infrastructure, is associated with a lower prevalence of youth smoking, fewer cigarettes smoked per day among smokers, and a faster decline in US smoking prevalence over time [20,21,22]. Low-income smokers may also be more likely to use and benefit from quitlines, which are funded by state TCF [23].

Although research suggests that differences exist in the relationship between tobacco control policies and smoking by income, few studies have confirmed or quantified these differences using national samples of adults, and gaps in the literature remain. Dinno and Glantz [24] used Tobacco Use Supplement to the Current Population Survey (TUS-CPS) data to examine the relationship between taxes, SFALs, and current smoking accounting for multiple socioeconomic factors, including income. The analysis did not find any differences in the relationship between either taxes or SFALs and current smoking by income [24]. This analysis only included one year of TUS-CPS data (2002), and the results are outdated. Numerous studies have examined the impact of SFALs and other tobacco control policies on smoking behavior in institutions commonly frequented by low-income individuals, such as homeless shelters and public housing [25]. However, it is not possible to generalize these results to the population level.

Regarding TCF, research has focused on one component of TCF, mass media campaigns, and suggests that the relationship between these interventions and smoking may not differ by socioeconomic status (SES), or that these policies may be less effective for low-SES populations [26,27,28]. However, this research only reveals information about mass media campaigns, not total TCF. Although research has examined the relationship between total TCF and smoking [20,21,22,29], existing publications did not test for differential relationships by income. Establishing whether differential effects exist for all three types of policies by income will help clarify whether these policies have the potential to narrow the income gap in smoking prevalence on a population level.

To our knowledge, this is the first national analysis to simultaneously examine variation in the relationship between SFALs, tobacco taxes, and TCF and adult current cigarette smoking by income over such a long time period. We improved upon previous research by converting income into poverty status because poverty may capture relative deprivation better than income. Establishing policy effects by poverty has the potential to influence local (city and county) and state tobacco control policies, inform the allocation of limited tobacco control funds, and reveal methods of combating income-based disparities in tobacco use [7,14,23,27]. We hypothesized that we would find a negative association between SFALs, tobacco taxes, TCF, and current smoking. We also expected that these policies would have a stronger association with current smoking among individuals living in poverty than those not living in poverty. We found that coverage by smoke-free laws, state tobacco taxes, and state tobacco control funding were all associated with lower odds of current cigarette smoking among adults. We also found differences in the relationship between smoke-free law coverage and tobacco control funding by poverty status.

## 2. Materials and Methods

### 2.1. Sample

We used data from the 1985 to 2015 supplements to the Current Population Survey (CPS), a monthly survey of the US civilian, non-institutionalized population [30]. CPS participants are selected by sampling large within-state geographies, then households. One household member completes the survey. All participants are also asked to complete a supplement containing specific content areas. State identifiers from the supplements were used to match state-level policy variables to individual data. For the 1980s, we used the 1985 Immunization and Smoking Survey supplement (administered to individuals ages 16 and older in approximately 71,000 households [31]) and the 1989 Smoking and Cardiovascular Disease Supplement (administered to individuals age 15 and older in approximately 56,000 households [32]) because these surveys provided current smoking status [33] and enabled us to establish trends in current smoking in the 1980s, before the introduction of many tobacco control policies in the 1990s [20]. Among participants who completed the CPS, the estimated response rate was 93% for each of these supplements.

We used all available years of individual data from the TUS-CPS (1992–1993, 1998–1999, 2000, 2001–2002, 2003, 2006–2007, 2010–2011, and 2014–2015). The TUS-CPS was administered to individuals ages 15 and older between 1992 and 2006 and to individuals 18 years and older between 2010 and 2015 in approximately 59,000 households per wave. [34] Response rates ranged from a low of 77.8% for 2014–2015 to a high of 88.0% for 1992–1993. [34,35,36,37,38] To examine adult smoking, analyses were restricted to individuals 18 years and older. We did not obtain Institutional Review Board approval for this study given that our study did not involve human subjects and our analysis was based on publicly available data obtained from the United States Census Bureau.

### 2.2. Outcome Variable

The outcome was individual-level current smoking status. The 1985 and 1989 supplements defined current smokers as having smoked at least 100 cigarettes in their lifetime and responding “yes” to “Do you smoke cigarettes now?”. The TUS-CPS (1992–2015) defined current smokers as having smoked at least 100 cigarettes in their lifetime and responding “every day” or “some days” (versus “not at all”) to “Do you now smoke cigarettes every day, some days, or not at all?”. All other participants were considered non-current smokers.

### 2.3. Independent Variables

#### 2.3.1. Primary Independent Variables

##### Poverty

We calculated poverty by dividing CPS self-reported household income by the poverty guideline, accounting for the number of household members, then dichotomizing the percentage into the categories of living in poverty (<138% of the poverty line) or not living in poverty (≥138% of the poverty line) [39]. We redefined poverty due to evidence that the poverty line is insufficient for measuring poverty [40,41,42] and should be increased [42]. Categorizing poverty using the poverty line may be too conservative to capture income-based health disparities [39,43]. We chose 138% of the poverty line because this is the cut-off for qualifying for Medicaid. Technically, the cut-off is 133% of the poverty line to qualify for Medicaid, but Medicaid applicants cannot count 5% of their incomes towards eligibility, thus making the cut-off 138% in actuality [44,45]. We used yearly national Social Security Association poverty guidelines for all states and the District of Columbia (DC), except for Hawaii and Alaska, for which we used these states’ guidelines [46].

##### SFALs

We obtained yearly information on the percentage of each state’s population covered by 100% workplace, restaurant, and bar SFALs from the American Nonsmokers’ Rights Foundation (ANRF) [47]. ANRF defines 100% smoke-free workplace, restaurant, and bar laws as prohibiting smoking in these venues, even in separately ventilated rooms, with one exemption—self-employed and single-employee workplaces or family-owned workplaces (all employees related to owner) not open to the public [48]. We used these laws to assign a value of 0 to 1 for SFAL coverage for each state-year (including DC). We assigned 0 to states with no 100% city, county, or state SFALs and 1 to states with a 100% state SFALs. For the remaining states, we matched county and city 100% SFALs to county and city population from the U.S. Census. We then summed the population covered by 100% SFALs and divided by the total population of the state, resulting in a proportion ranging from 0 to 1 for SFAL coverage.

Because the effects of SFALs can be complex, we tested two methods of coding SFALs: (1) SFAL-All (the portion of the state’s population covered by 100% smoke-free workplace, restaurant, and bar laws) or (2) SFAL workplace (portion of population covered by 100% smoke-free workplace laws; SFAL-WP) and SFAL restaurant/bar (portion of population covered by 100% smoke-free restaurant and/or bar laws; SFAL-RB) to account for differences in the relationship between workplace and restaurant/bar SFALs and smoking behavior [49,50]. Models either included SFAL-All or SFAL-WP and SFAL-RB. The mean correlation between SFAL-WP and SFAL-RB across all years of data was moderate at 0.77. As a result, after running the models that included both variables, we ran separate models that only included SFAL-WP or SFAL-RB to ensure that collinearity was not a problem.

##### Tobacco Tax

We matched state per-pack cigarette taxes from the Tax Burden of Tobacco (TBOT) [51] to participant state-year. We used the Consumer Price Index (CPI) [52] to adjust for inflation and express taxes in 2015 dollars.

##### Per Capita State Tobacco Control Funding

We also matched state per-capita tobacco control funding (TCF) in inflation-adjusted 2015 dollars [52] from RTI International’s internal TCF database [20,21,29,53,54] to each participant by state-year. These funds include federal and state funding for tobacco control, including excise taxes earmarked for tobacco control, state settlement and Master Settlement Agreement sources, funding from the Centers for Disease Control and Prevention, and other state appropriations for tobacco control, with a great deal of variability in the amount allocated to each of these activities across states [21]. We calculated funding amounts for each year at a 5% discount to reflect that all funds may not have been immediately available even after being allocated to the state.

Individual-level demographic variables served as secondary independent variables. We obtained individual demographic data from the CPS, including age (linear and quadratic terms to account for nonlinear relationship with smoking), race/ethnicity (non-Hispanic white (reference), non-Hispanic black, Hispanic, or non-Hispanic other), education (never attended high school, attended high school but did not graduate, high school degree/General Education Diploma (GED; reference), some college education, or college degree or greater education), marital status (married (reference), divorced, widowed, separated, or never married), and employment status (employed (reference), unemployed, or not in the labor force).

### 2.4. Analysis

We conducted all analyses in Stata 15.1 (StataCorp, Lakeway, TX, USA), adjusting for weights provided by the Bureau of Labor Statistics for each year of data collection. Means and frequencies were used to describe covariates for the overall sample and by poverty. Simple F-tests of trend examined changes in the characteristics of the sample over time, and adjusted Wald tests produced p-values for comparisons of mean values of covariates (across all years) by poverty. Next, we constructed logistic regression models. Because income was missing for 8.87% of the analytical sample, multiple imputation was used to estimate missing values of poverty (based on participants’ gender, age, race/ethnicity, education, household size, marital status, employment, and state-year) in all regression models using the Stata “mi estimate” command. Diagnostics for the final models suggested low variance attributable to missing income (3%) and high efficiency (99.7%) using 10 datasets for imputation. After imputation, we created interaction terms for poverty and SFALs, poverty and tax, and poverty and TCF and added these terms to logistic regression models to quantify differential effects of these policies on smoking by poverty. We interpret these results with and without interaction terms. Using the Stata “margins” command [55], for significant interaction terms, we graphed the relationship between the adjusted predictions of smoking (e.g., model-predicted smoking) and the policy variables, stratified by poverty status. All regression models included state fixed effects, year as a fixed effect, and time-varying state fixed effects (state by year interaction). Of the 2,244,437 adults (age 18 and older) who completed the CPS supplements, 2,014,811 (89.77%) had non-missing values for all variables of interest (except income, which was imputed) and were included in the analysis.

A sensitivity analysis dropped data from the 1985 and 1989 surveys from our models to determine whether variation in the definition of current smoking between the 1985 and 1989 surveys and the TUS-CPS surveys influenced our results. A second sensitivity analysis assessed whether the effect estimates in the model changed when participants with missing income were dropped from the analysis. A third sensitivity analysis examined whether the results of the analysis changed if the cut-off for poverty was set at 150% of the poverty line instead of 138%. We chose 150% because this value separated individuals in the bottom 20% of the income distribution from the rest of the sample, and 20% is a cut-off commonly used to study income inequality [56].

## 3. Results

### 3.1. Descriptive Statistics

The proportion of current smokers in the sample changed greatly over time. Current smoking decreased from 29.15% (SD = 45.26%) in 1985 to 15.15% (SD = 36.13%) in 2011 (*p* < 0.001). In parallel, tobacco control policies changed over time. SFALs increased from 0% to 45.19% (SD = 45.56%; *p* < 0.001) for workplace, restaurant, and bar laws, from 0% to 62.13% (SD = 41.45%) for workplace laws (*p* < 0.001), and from 0% to 75.06% (SD = 39.18%) for restaurants or bar laws (*p* < 0.001). Mean state per-pack cigarette tax increased from $0.70 (SD = $0.13) to $2.64 (SD = $1.12). TCF increased from less than $0.01 (SD = $0.01) to $2.48 (SD = $1.77).

The demographics of the sample changed over time, including increases in mean age (*p* < 0.001), racial and ethnic diversity (*p* < 0.001 for all categories), the percentage of participants living below 138% of the poverty line (*p* < 0.001), education (*p* < 0.001 for some college and college graduate or beyond), the proportion that was divorced and never married (both *p* < 0.001), and unemployment (*p* < 0.001).

### 3.2. Bivariate Statistics

The proportion of current smokers was significantly higher among adults living below 138% of the poverty line (27.8%) than those living at or above it (18.56%, *p* < 0.001; Table 1). Smokers living in poverty were significantly less likely to be covered by SFAL-All (12.26% versus 13.65%, *p* < 0.001), SFAL-WP (24.59% versus 25.83%, *p* < 0.001), and SFAL-RB (18.41% versus 19.36%, *p* < 0.001) than participants not living in poverty. Participants living in poverty also experienced, on average, lower state tobacco taxes ($1.36 versus $1.43, *p* < 0.001) and state TCF ($13.94 versus $14.87, *p* < 0.001) than participants not living in poverty. There were also differences in demographics by poverty, with lower age (*p* < 0.001), greater racial and ethnic diversity (all groups *p* < 0.001), lower education (*p* < 0.001 for all categories), less marriage (*p* < 0.001), and lower employment levels (*p* < 0.001) among those living in poverty.

### 3.3. Logistic Regression Models

We used two different model specifications (listed below by specification) to assess the relationship between policies and current smoking by poverty. The overall models assume the same effect across poverty; the interaction models allowed effects to differ across poverty.

#### 3.3.1. Overall Models

##### SFAL-All Coverage

In the SFAL-All model without interactions (Table 2, Model 1), living in poverty was significantly (and positively) associated with current smoking (Coeff = 0.32, 95% CI: 0.30, 0.33), but SFAL-All was not (Coeff = −0.01, 95% CI: −0.04, 0.01). Current smoking was lower for individuals living in states with higher taxes (Coeff = −0.06, 95% CI: −0.07, −0.05) and higher TCF (Coeff = −0.01, 95% CI: −0.01, −0.002). Age, age squared, race, education, marital status, and employment were all significantly associated with current smoking (all *p* < 0.001) with the exception of smokers with less than a high school education (Coeff = 0.02, 95% CI: −0.001, 0.04) compared to smokers who were high school graduates or had a GED. 

##### SFAL-WP and SFAL-RB Coverage

In the SFAL-WP and SFAL-RB model without interactions (Table 2, Model 2), SFAL-WP was not significantly associated with current smoking (Coeff = 0.02, 95% CI: −0.01, 0.05), but SFAL-RB was (Coeff = −0.08, 95% CI: −0.11, −0.06). The direction and significance of all other variables were the same as in the SFAL-All model (Table 2, Model 1) except that TCF was no longer significant (Coeff = −0.003, 95% CI: −0.01, 0.001). To confirm that collinearity was not a problem in these models (Models 2 and 4, Table 2), we also modeled SFAL-WP and SFAL-RB in separate models [57]. Given that the effect estimates were similar when these variables were modeled separately as when both variables were included in the model, we concluded that collinearity was not a problem and included both variables [57].

#### 3.3.2. Models Containing Interactions

##### SFAL-All Coverage

**Figure 1:**
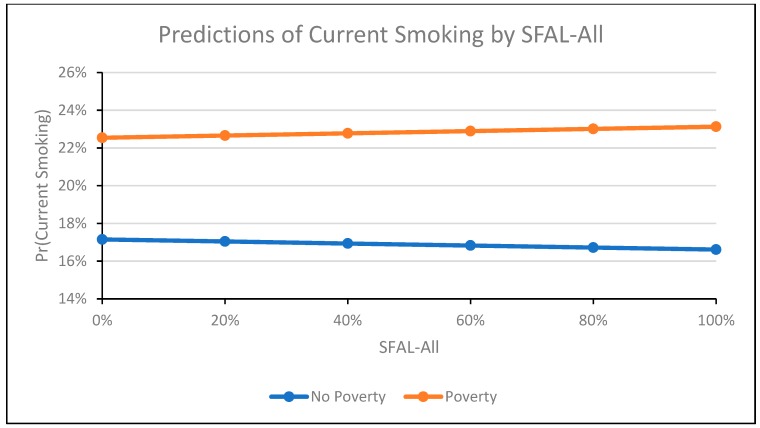
This figure depicts the probability of individual-level adult current smoking in the 1985−2015 supplements to the U.S. Current Population Survey by percent of state population covered by workplace, bar, and restaurant smoke-free laws (SFAL-All), stratified by individual poverty status (living < 138% of poverty line versus living ≥138% of poverty line). The figure excludes participants with missing values for variables of interest and was produced using the “margins” command in Stata.

In the SFAL-All model (Table 2, Model 3), interaction terms for poverty and tax (Coeff = −0.01, 95% CI: −0.03, 0.02) and poverty and TCF (Coeff = −0.002, 95% CI: −0.01, 0.01) were not significant, indicating that the relationship between tax and smoking, and TCF and smoking, did not differ significantly by poverty. The interaction term for poverty and SFAL-All was significant and positive (Coeff = 0.08, 95% CI: 0.02, 0.13). The relationship between SFAL-All and predicted smoking was positive among participants living in poverty and negative among participants not living in poverty (Figure 1). SFAL-All coverage remained associated with a lower proportion of current smokers (Coeff = −0.03, 95% CI: −0.06, −0.002) and became significant. The directionality and significance of all other covariates remained the same as in the SFAL-All model that did not contain interactions (Table 2, Model 1). 

##### SFAL-WP and SFAL-RB Coverage

**Figure 2:**
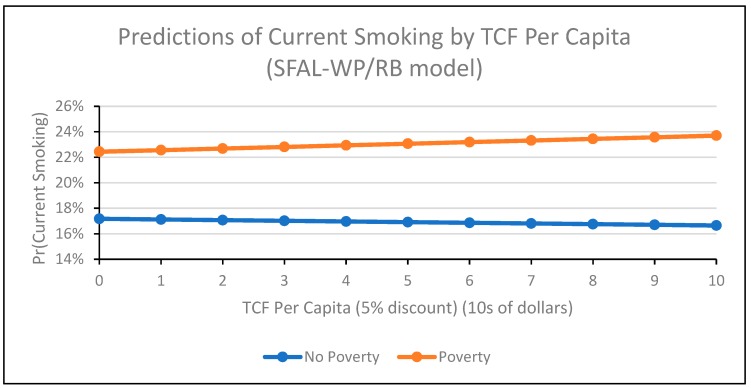
This figure depicts the probability of individual-level adult current smoking in the 1985−2015 supplements to the U.S. Current Population Survey by state tobacco control funding (TCF), stratified by individual-level poverty status (living <138% of poverty line versus living ≥138% of poverty line). Smoke-free law coverage is modeled as percent of state population covered by workplace laws (SFAL-WP) and percent covered by restaurant and/or bar laws (SFAL-RB), and the figure excludes participants with missing values for variables of interest. The figure was produced using the “margins” command in Stata.

**Figure 3:**
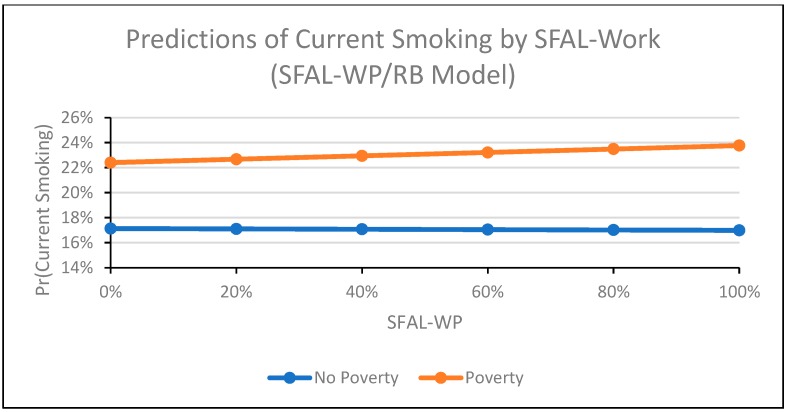
This figure depicts the probability of individual-level adult current smoking in the 1985−2015 supplements to the U.S. Current Population Survey by percent of state population covered by workplace smoke-free laws, stratified by poverty status (living <138% of poverty line versus living ≥138% of poverty line). Smoke-free law coverage is modeled as the percent of state population covered by workplace laws and percent covered by restaurant and/or bar laws, and the figure excludes participants with missing values for variables of interest. The figure was produced using the “margins” command in Stata.

**Figure 4:**
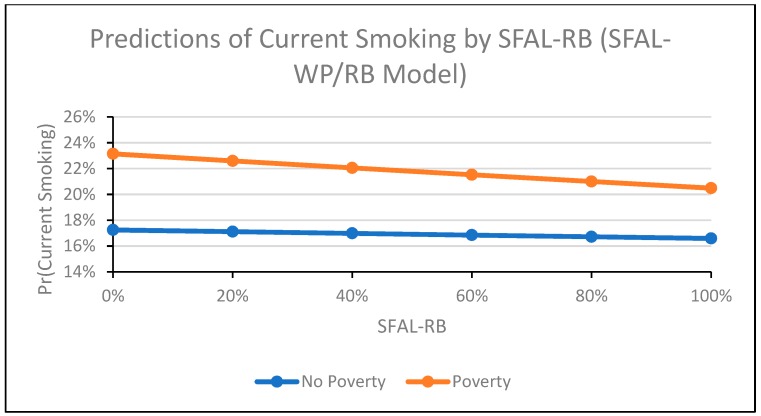
This figure depicts the probability of individual-level adult current smoking in the 1985−2015 supplements to the U.S. Current Population Survey by percent of state population covered by restaurant and/or bar smoke-free laws, stratified by poverty status (living <138% of poverty line versus living ≥138% of poverty line). Smoke-free law coverage is modeled as the percent of state population covered by workplace laws and percent covered by restaurant and/or bar laws, and the figure excludes participants with missing values for variables of interest. The figure was produced using the “margins” command in Stata.

In the SFAL-WP and SFAL-RB model (Table 2, Model 4), the interaction term for tax and poverty approached significance (Coeff = 0.02, 95% CI: −0.00, 0.04; *p* = 0.050), suggesting that the relationship between tax and smoking may differ by poverty. The interaction term for poverty and TCF was significant and positive (Coeff = 0.01, 95% CI: 0.00, 0.02). The relationship between TCF and smoking was positive among participants living in poverty and negative among participants not living in poverty (Figure 2). The interaction term for poverty and SFAL-WP was significant and positive (Coeff = 0.09, 95% CI: 0.03, 0.15; Figure 3), and the interaction term for poverty and SFAL-RB was significant and negative (Coeff = −0.14, 95% CI: −0.19, −0.09; Figure 4). For workplace laws (Figure 3), the slope of the relationship between SFAL-WP coverage and smoking was positive among those living in poverty and flat (close to 0) for those not living in poverty. The relationship between SFAL-RB coverage (Figure 4) and smoking was negative regardless of poverty, but this relationship was stronger among participants living in poverty compared to those not living in poverty. The direction and significance of all other covariates remained the same in the SFAL-WP and SFAL-RB model (Table 2, Model 4) as when the interactions were not included in the model (Table 2, Model 2).

#### 3.3.3. Sensitivity Analyses

The first sensitivity analysis, which excluded the first two years of data, resulted in two differences in the results for the models that contained interactions. TCF was nonsignificant in the SFAL-All model (Coeff = −0.002, 95% CI: −0.01, 0.003; Supplementary Table S1, Model 1) and in the SFAL-WP and SFAL-RB model (Coeff = −0.003, 95% CI: −0.01, 0.002; Supplementary Table S1, Model 2). In addition, the interaction term for TCF by poverty became nonsignificant in the SFAL-WP and SFAL-RB model (Coeff = −0.003, 95% CI: −0.01, 0.004). The second sensitivity analysis dropped all individuals with missing income from the analyses conducted with interaction terms. The only resulting difference was that TCF was no longer significant for either the SFAL-All model (Coeff: −0.01, 95% CI: −0.01, 0.0001; Supplementary Table S1, Model 3) or the SFAL-WP and SFAL-RB model (Coeff: −0.004, 95% CI: −0.01, 0.001; Supplementary Table S1, Model 4). The third sensitivity analysis, which changed the cut-off for poverty to 150% of the poverty line, also resulted in similar findings. For the models that included interaction terms, there were no differences in the SFAL-All model (Supplementary Table S1, Model 5). The only difference was that the interaction for tax by poverty did not approach significance in the SFAL-WP and SFAL-RB model (Coeff= 0.01, −0.01, 0.03; Supplementary Table S1, Model 6).

## 4. Discussion

This analysis found a significant negative relationship between percent of the county covered by bar and/or restaurant SFALs and percent of the county covered by workplace, bar, and/or restaurant laws and current smoking, but not percent of the county covered by workplace laws and smoking. We also found that coverage by taxes and TCF were associated with a significantly lower proportion of current smokers. This analysis confirmed the established link between poverty and cigarette smoking in the U.S. [1,58]. In addition, this analysis confirmed existing literature regarding the potential benefits of SFALs [1], taxes [10,58], and TCF [1,20] on current U.S. adult smoking prevalence.

Overall, our findings are generally consistent with the established literature. Our finding of a nonsignificant association between workplace laws and smoking status is consistent with existing studies of workplace laws and youth and adult smoking status [49,59]. Workplace SFALs generally pass before restaurant and bar SFALs, and the latter are more likely to reflect social norms change [60].

We also found evidence that the relationship between tobacco control policies and current smoking differed by poverty for SFALs and TCF, and perhaps for cigarette tax, confirming the need for model specifications such as interaction terms to test for differential effects of SFALs and TCF on tobacco use by poverty [61]. Our finding of a difference in the relationship between SFALs and smoking by poverty (regardless of how SFAL coverage was measured) is consistent with evidence from other research that SFALs may be more effective at reducing SHS exposure and promoting smoking cessation among individuals in higher occupational grades [14,62,63], a category that is unlikely to include individuals living close to or below the poverty line. Similarly, research demonstrates that, even after workplace SFALs have passed, staff in lower occupational grades reported higher levels of SHS [63] and had a greater probability of continuing to smoke than workers in higher occupational grades [62]. Our finding of variation in the relationship between coverage by state bar and/or restaurant SFALs and smoking by poverty is also consistent with literature, which suggests that bar and restaurant SFALs may be particularly effective for low-income workers because they are more likely to be employed in these venues [64]. In addition, our finding of a significant interaction for TCF by poverty when we coded SFALs as workplace and restaurant/bar is also consistent with existing literature that supports potential differential effects of TCF funding by income [6].

In contrast to literature suggesting that low-income smokers are more price sensitive than high income smokers [13,14,15], we did not find evidence for differences in the relationship between tax and smoking by poverty. Although low-income and high-income smokers may, on average, spend the same amount per pack on cigarettes, higher cigarette taxes tend to place a larger financial burden on low-income smokers than on high-income smokers [12], potentially making them more sensitive to price changes caused by tax increases [13,14,15]. However, price minimizing behaviors (e.g., buying cartons instead of packs, tax evasion) may offset the effects of taxes on smoking among low-income individuals [9,13].

### Limitations

This analysis has several limitations. Our definition of smoking varied slightly over time because we used two non-TUS-CPS supplements. However, because this practice was necessary to establish baseline current smoking before many tobacco control policies were enacted in the 1990s [20] and the sensitivity analyses that excluded these years produced similar results, we view this choice as a strength. Another limitation is that, for several reasons, one of which is our use of data from repeated cross-sectional surveys (not longitudinal data), the causal effect of policies on individual behavior change cannot be determined from our results. In addition, given that we utilized a non-linear regression model, interpretation of the interaction terms is complicated. We took the simple approach of interpreting the directionality of the coefficient of the interaction terms. However, the interaction effect may differ at different levels of other variables in the model. In addition, we were unable to account for potential three-way interactions between policy, poverty, and time; our attempts to model changes in these interactions over time created model instability and produced unrealistic effect estimates. Lastly, we did not have access to funding amounts for the different programs that compose TCF (e.g., mass media campaigns, quitlines), and, as a result, we could not determine which components of TCF were driving the observed effects.

## 5. Conclusions

Our results suggest that SFALs, taxes, and TCF are all important elements for combatting adult current cigarette smoking in the United States regardless of poverty status. In addition, our research found differences in the relationship between SFALs and TCF and current smoking by poverty status. Closing gaps in existing smoke-free law coverage and increasing state TCF may be particularly beneficial for smokers living close to or below the poverty line. Addressing disparities by creating targeted policies that are effective for low-income individuals can help reduce the burden of tobacco among underserved communities.

## Figures and Tables

**Table 1:** Outcome and covariates for the overall U.S. Census Current Population Survey supplement sample (1985–2015) and by poverty status ^1^.

Variable Name	Equal to or above 138% of Poverty Line *n* = 1,683,311	Below 138% of Poverty Line *n* = 431,711	Total *n* = 2,070,022	*p* Value
**Current Smoker, % (SE)**				
Yes	18.56 (0.03)	27.82 (0.07)	20.49 (0.03)	<0.0001
**Primary predictor variables, Mean (SE)**				
Smoke-free law coverage (SFALs) ^2^				
% state covered by workplace, bar, and restaurant laws (All)	13.65 (0.03)	12.26 (0.05)	13.36 (0.02)	<0.0001
% state covered by workplace laws (WP)	25.83 (0.03)	24.59 (0.06)	25.57 (0.03)	<0.0001
% state covered by restaurant and/or bar laws (RB)	19.36 (0.03)	18.41 (0.05)	19.16 (0.02)	<0.0001
Per-pack state cigarette tax ($) ^3^	1.43 (0.001)	1.36 (0.001)	1.42 (0.001)	<0.0001
Per capita tobacco control funding - 5% discount ($) ^4^	14.87 (0.02)	13.94 (0.03)	14.68 (0.01)	<0.0001
**Covariates, Mean (SE)**				
Age	45.92 (0.01)	45.37 (0.03)	45.80 (0.01)	<0.0001
Income ($ ^5^)	84,458.34 (39.91)	16,391.33 (12.27)	70,262.71 (37.11)	<0.0001
Covariates, % (SE)				
Race/ethnicity				
Non-Hispanic white	81.57 (0.03)	59.08 (0.07)	76.88 (0.03)	<0.0001
Non-Hispanic black	6.90 (0.02)	16.79 (0.06)	8.96 (0.02)	<0.0001
Hispanic	6.63 (0.02)	17.54 (0.06)	8.91 (0.02)	<0.0001
Non-Hispanic other	4.89 (0.02)	6.58 (0.04)	5.24 (0.02)	<0.0001
Educational attainment				
No high school	3.51 (0.02)	17.39 (0.06)	6.41 (0.02)	<0.0001
High school dropout	7.12 (0.02)	20.72 (0.07)	9.96 (0.02)	<0.0001
High school graduate/ General Education Diploma (GED)	32.42 (0.04)	35.68 (0.08)	33.10 (0.03)	<0.0001
Some college	28.08 (0.04)	19.50 (0.06)	26.29 (0.03)	<0.0001
College graduate	28.87 (0.04)	6.71 (0.04)	24.25 (0.03)	<0.0001
Marital status				
Married	63.68 (0.04)	40.03 (0.07)	58.75 (0.03)	<0.0001
Divorced	9.23 (0.02)	13.26 (0.05)	10.07 (0.02)	<0.0001
Widowed	5.49 (0.02)	12.33 (0.05)	6.91 (0.02)	<0.0001
Separated	1.54 (0.01)	4.47 (0.03)	2.15 (0.01)	<0.0001
Never married	20.06 (0.03)	29.91 (0.07)	22.12 (0.03)	<0.0001
Employment status				
Working	69.91 (0.04)	42.21 (0.08)	64.13 (0.03)	<0.0001
Unemployed	2.78 (0.01)	7.51 (0.04)	3.77 (0.01)	<0.0001
Not in labor force	27.31 (0.03)	50.28 (0.08)	32.10 (0.03)	<0.0001

^1^ Poverty was defined as living below 138% of the poverty line versus living at or above 138% of the poverty line. ^2^ Includes 100% workplace, restaurant, and bar laws. Information obtained from the Americans for Nonsmokers Rights Foundation. ^3^ Obtained from the Tax Burden of Tobacco. Adjusted for inflation to 2015 dollars using the Consumer Price Index (CPI) [52]. ^4^ State-level funding obtained from RTI International’s database, calculated at a 5% discount. ^5^ Adjusted for inflation to 2015 dollars using the CPI.

**Table 2:** Multivariable logistic regression models of current prevalence of cigarette smoking regressed on poverty status ^1^ and tobacco control policies with and without interaction terms for poverty and tobacco control policies, using data from supplements to the U.S. Census Current Population Survey (1985–2015).

Variable Name	Without Interaction Terms		With Interaction Terms	
	Model 1	Model 2	Model 3	Model 4
	Coeff (95% CI)	Coeff (95% CI)	Coeff (95% CI)	Coeff (95% CI)
Poverty (<138% of poverty line)	0.32 (0.30, 0.33)	0.32 (0.30, 0.33)	0.32 (0.30, 0.34)	0.30 (0.28, 0.32)
Smoke-free law coverage (SFALs) ^2^				
% state covered by workplace, bar, and restaurant laws (All)	−0.01 (−0.04, 0.01)	-	−0.03 (−0.06, −0.002)	-
% state covered by workplace laws (WP)	-	0.02 (−0.01, 0.05)	-	−0.002 (−0.04, 0.03)
% of state covered by restaurant and/or bar laws (RB)	-	−0.08 (−0.11, −0.06)	-	−0.05 (−0.08, −0.02)
Poverty by SFAL-All	-	-	0.08 (0.02, 0.13)	-
Poverty by SFAL-WP	-	-	-	0.09 (0.03, 0.15)
Poverty by SFAL-RB	-	-	-	−0.14 (−0.19, −0.09)
Per-pack state cigarette tax ($) ^3^	−0.06 (−0.07, −0.05)	−0.05 (−0.06, −0.04)	−0.06 (−0.07, −0.05)	−0.05 (−0.06, −0.04)
Poverty by Tax	-	-	−0.01 (−0.03, 0.02)	0.02 (−0.00, 0.04)
Per capita tobacco control funding - 5% discount ($) ^4^	−0.01 (−0.01, −0.002)	−0.003 (−0.01, 0.001)	−0.01 (−0.01, −0.001)	−0.01 (−0.01, −0.00)
Poverty by TCF	-	-	−0.002 (−0.01, 0.01)	0.01 (0.00, 0.02)
Age	0.11 (0.11, 0.11)	0.11 (0.11, 0.11)	0.11 (0.11, 0.11)	0.11 (0.11, 0.11)
Age ²	−0.001 (−0.001, −0.001)	−0.001 (−0.001, −0.001)	−0.001 (−0.001, -0.001)	−0.001 (−0.001, −0.001)
Race/ethnicity				
Non-Hispanic white	Ref	Ref	Ref	Ref
Non-Hispanic black	−0.50 (−0.51, −0.48)	−0.50 (−0.51, −0.48)	−0.50 (−0.51, −0.48)	−0.50 (−0.51, −0.48)
Hispanic	−0.90 (−0.92, −0.88)	−0.90 (−0.92, −0.88)	−0.90 (−0.92, −0.88)	−0.90 (−0.91, −0.88)
Non-Hispanic other	−0.36 (−0.38, −0.33)	−0.36 (−0.38, −0.33)	−0.36 (−0.38, −0.33)	−0.36 (−0.38, −0.33)
Educational attainment				
No high school	0.02 (−0.001, 0.04)	0.02 (−0.001, 0.04)	0.02 (−0.001, 0.04)	0.02 (−0.00, 0.04)
High school dropout	0.42 (0.41, 0.43)	0.42 (0.41, 0.43)	0.42 (0.41, 0.43)	0.42 (0.41, 0.43)
High school graduate/General Education Diploma (GED)	Ref	Ref	Ref	Ref
Some college	−0.36 (−0.37, −0.35)	−0.36 (−0.37, −0.35)	−0.36 (−0.37, −0.35)	−0.36 (−0.37, −0.35)
College graduate	−1.22 (−1.23, −1.20)	−1.22 (−1.23, −1.20)	−1.22 (−1.23, −1.20)	−1.22 (−1.23, −1.20)
Marital Status				
Married	Ref	Ref	Ref	Ref
Divorced	0.74 (0.73, 0.75)	0.74 (0.73, 0.75)	0.74 (0.73, 0.75)	0.74 (0.72, 0.75)
Widowed	0.41 (0.39, 0.43)	0.41 (0.39, 0.43)	0.41 (0.39, 0.43)	0.41 (0.39, 0.43)
Separated	0.74 (0.71, 0.76)	0.74 (0.71, 0.76)	0.74 (0.71, 0.76)	0.74 (0.71, 0.76)
Never married	0.33 (0.32, 0.35)	0.33 (0.32, 0.35)	0.33 (0.32, 0.35)	0.33 (0.32, 0.35)
Employment				
Working	Ref	Ref	Ref	Ref
Unemployed	0.45 (0.43, 0.47)	0.45 (0.43, 0.47)	0.45 (0.43, 0.47)	0.45 (0.43, 0.47)
Not in labor force	−0.09 (−0.10, −0.10)	−0.09 (−0.10, −0.08)	−0.09 (−0.10, −0.08)	−0.09 (−0.10, −0.10)

^1^ Poverty was defined as living below 138% of the poverty line versus living at or above 138% of the poverty line. ^2^ Includes 100% workplace, restaurant, and bar laws. Information obtained from the Americans for Nonsmokers Rights Foundation. ^3^ Obtained from the Tax Burden of Tobacco. Adjusted for inflation to 2015 dollars using the Consumer Price Index [52]. ^4^ State-level funding obtained from RTI International’s database, calculated at a 5% discount.

## Supplementary Materials

The following are available online at https://www.mdpi.com/1660-4601/16/21/4130/s1, Table S1: results of sensitivity analyses for survey year and imputed income for multivariable logistic regression models with interaction terms using data from 1985–2015 supplements to the U.S. Census Current Population Survey with imputed income and defining poverty as 138% of the poverty line unless otherwise noted.
