# Supplementary material for: Differential Relationship between Tobacco Control Policies and U.S. Adult Current Smoking by Poverty
Source: Int J Environ Res Public Health. 2019 Oct 26;16(21):4130. doi: 10.3390/ijerph16214130 (PMC6862047; doi:10.3390/ijerph16214130)
Supplement: Supplementary file 1 [file ijerph-16-04130-s001.pdf]

Supplementary Table 1. Results of sensitivity analyses for survey year and imputed income for multivariable logistic regression models with interaction terms using data from 1985-2015 supplements to the U.S. Census Current Population Survey with imputed income and defining poverty as 138% of the poverty line unless otherwise noted).

	**Data from only 1992-2015 supplements**		**Dropped participants with missing income**		**Alternate definition of poverty as 150% of poverty line**	
	**Model 1**	**Model 2**	**Model 3**	**Model 4**	**Model 5**	**Model 6**
	**Coeff (95%CI)**	**Coeff (95%CI)**	**Coeff (95%CI)**	**Coeff (95%CI)**	**Coeff (95%CI)**	**Coeff (95%CI)**
Poverty	0.33 (0.31, 0.36)[1]	0.31 (0.29, 0.33)[1]	0.35 (0.33, 0.38)[1]	0.33 (0.31, 0.35)[1]	0.32 (0.30, 0.34)	0.30 (0.28, 0.32)
Smoke-free law coverage (SFALs)[2]						
% state covered by workplace, bar, and restaurant laws (All)	-0.04 (-0.07, -0.01)	-	-0.04 (-0.07, -0.01)	-	-0.04 (-0.07, -0.01)	-
% state covered by workplace laws (WP)	-	-0.02 (-0.05, 0.02)	-	-0.01 (-0.04, 0.02)	-	-0.01 (-0.04, 0.03)
% of state covered by restaurant and/or bar laws (RB)	-	-0.04 (-0.07, -0.01)	-	-0.04 (-0.07, -0.01)	-	-0.05 (-0.08, -0.02)
Poverty * SFAL-All	0.08 (0.02, 0.13)	-	0.07 (0.01, 0.13)	-	0.09 (0.04, 0.15)	-
Poverty * SFAL-WP	-	0.09 (0.02, 0.15)	-	0.09 (0.03, 0.15)	-	0.09 (0.03, 0.15)
Poverty * SFAL-RB	-	-0.14 (-0.19, -0.09)	-	-0.15 (-0.20, -0.10)	-	-0.13 (-0.17, -0.08)
Per-pack state cigarette tax ($)[3]	-0.06 (-0.07, -0.05)	-0.06 (-0.07, -0.04)	-0.07 (-0.08, -0.05)	-0.06 (-0.07, -0.05)	-0.06 (-0.07, -0.05)	-0.05 (-0.07, -0.04)
Poverty * Tax	-0.01 (-0.03, 0.01)	0.01 (-0.01, 0.03)	-0.01 (-0.03, 0.01)	0.02 (-0.01, 0.04)	-0.01 (-0.03, 0.01)	0.01 (-0.01, 0.03)
Per capita tobacco control funding- 5% discount ($)[4]	-0.002 (-0.01, 0.003)	-0.003 (-0.01, 0.002)	-0.01 (-0.01, 0.0001)	-0.004 (-0.01, 0.001)	-0.01 (-0.01, -0.001)	-0.01 (-0.01, -0.0002)
Poverty * TCF	-0.003 (-0.01, 0.004)	0.01 (-0.001, 0.01)	0.001 (-0.01, 0.01)	0.01 (0.003, 0.02)	-0.0003 (-0.01, 0.01)	0.01 (0.001, 0.02)
Age	0.11 (0.11, 0.12)	0.11 (0.11, 0.12)	0.11 (0.11, 0.12)	0.11 (0.11, 0.12)	0.11 (0.11, 0.11)	0.11 (0.11, 0.11)

Age²	-0.001 (-0.001, -0.001)	-0.001 (-0.001, -0.001)	-0.001 (-0.001, -0.001)	-0.001 (-0.001, -0.001)	-0.001 (-0.001, -0.001)	-0.001 (-0.001, -0.001)
Race						
Non-Hispanic white	*Ref*	*Ref*	*Ref*	*Ref*	*Ref*	*Ref*
Non-Hispanic black	-0.54 (-0.55, -0.52)	-0.54 (-0.55, -0.52)	-0.50 (-0.52, -0.49)	-0.50 (-0.52, -0.49)	-0.50 (-0.52, -0.49)	-0.50 (-0.52, -0.49)
Hispanic	-0.92 (-0.93, -0.90)	-0.91 (-0.93, -0.89)	-0.90 (-0.92, -0.88)	-0.90 (-0.92, -0.88)	-0.90 (-0.92, -0.88)	-0.90 (-0.92, -0.88)
Non-Hispanic other	-0.35 (-0.38, -0.33)	-0.35 (-0.38, -0.33)	-0.35 (-0.37, -0.32)	-0.35 (-0.37, -0.32)	-0.36 (-0.38, -0.34)	-0.36 (-0.38, -0.34)
Educational attainment						
No high school	-0.02 (-0.05, -0.002)	-0.02 (-0.04, -0.002)	0.01 (-0.01, 0.03)	0.02 (-0.01, 0.04)	0.02 (-0.002, 0.04)	0.02 (-0.001, 0.04)
High school dropout	0.41 (0.39, 0.42)	0.41 (0.39, 0.42)	0.42 (0.40, 0.43)	0.42 (0.40, 0.43)	0.42 (0.41, 0.43)	0.42 (0.41, 0.43)
High school graduate	*Ref*	*Ref*	*Ref*	*Ref*	*Ref*	*Ref*
Some college	-0.37 (-0.38, -0.36)	-0.37 (-0.38, -0.36)	-0.37 (-0.38, -0.36)	-0.37 (-0.38, -0.36)	-0.36 (-0.37, -0.35)	-0.36 (-0.37, -0.35)
College graduate	-1.24 (-1.26, -1.23)	-1.24 (-1.26, -1.23)	-1.23 (-1.24, -1.22)	-1.23 (-1.24, -1.22)	-1.21 (-1.22, -1.20)	-1.21 (-1.22, -1.20)
Marital Status						
Married	*Ref*	*Ref*	*Ref*	*Ref*	*Ref*	*Ref*
Divorced	0.75 (0.74, 0.76)	0.75 (0.74, 0.76)	0.73 (0.72, 0.75)	0.73 (0.72, 0.74)	0.74 (0.73, 0.75)	0.74 (0.72, 0.75)
Widowed	0.43 (0.41, 0.45)	0.43 (0.41, 0.45)	0.40 (0.38, 0.43)	0.40 (0.38, 0.43)	0.41 (0.39, 0.43)	0.41 (0.39, 0.43)
Separated	0.75 (0.73, 0.78)	0.75 (0.72, 0.78)	0.73 (0.70, 0.75)	0.73 (0.70, 0.75)	0.74 (0.711, 0.76)	0.74 (0.71, 0.76)
Never married	0.37 (0.35, 0.38)	0.36 (0.35, 0.38)	0.34 (0.32, 0.35)	0.34 (0.32, 0.35)	0.34 (0.32, 0.35)	0.34 (0.32, 0.35)
Employment						
Working	*Ref*	*Ref*	*Ref*	*Ref*	*Ref*	*Ref*
Unemployed	0.46 (0.44, 0.48)	0.46 (0.44, 0.48)	0.44 (0.42, 0.46)	0.44 (0.42, 0.46)	0.45 (0.43, 0.47)	0.45 (0.43, 0.47)
Not in labor force	-0.07 (-0.09, -0.06)	-0.07 (-0.09, -0.06)	-0.09 (-0.10, -0.08)	-0.09 (-0.10, -0.08)	-0.09 (-0.10, -0.08)	-0.09 (-0.10, -0.08)

1 Poverty was defined as living below 138% of the poverty line versus living at or above 138% of the poverty line. 2 Includes 100% workplace, restaurant, and bar laws. Information obtained from the Americans for Nonsmokers Rights Foundation. 3 Obtained from the Tax Burden of Tobacco. Adjusted for inflation to 2015 dollars using the Consumer Price Index [52]. 4 State-level funding obtained from RTI International's database, calculated at a 5% discount.
